# Supplementary material for: Nile basin flow regimes under 21st century climate variability
Source: Commun Earth Environ. 2025 Nov 10;6(1):880. doi: 10.1038/s43247-025-02813-0 (PMC12602362; doi:10.1038/s43247-025-02813-0)
Supplement: Supplementary file 1 — Supplementary Information [file 43247_2025_2813_MOESM1_ESM.docx]

Nile basin flow regimes under 21^st^ century climate variability

Hesham Elhaddad^1,2^, Mohamed Sultan^1^, Eugene Yan^3^, Duc Tran^4^, Hugo E. Torres-Uribe^3^, Hadi Karimi^1^,

1 Department of Geological and Environmental Sciences, Western Michigan University, Kalamazoo, MI 49008, USA

2 Geodynamics Department, National Research Institute of Astronomy and Geophysics (NRIAG), Helwan, Cairo 11421, Egypt

3 Environmental Science Division, Argonne National Laboratory, Argonne, IL 60439, USA

4 Department of Civil and Environmental Engineering, The University of Virginia, Charlottesville, VA 22903, USA

Table of Contents

[Supplementary Text 1: 2](#_Toc209812195)

[Supplementary Text 2: 3](#_Toc209812196)

[Supplementary Text 3: 3](#_Toc209812197)

[Supplementary Text 4: 4](#_Toc209812198)

[Supplementary Figures 5](#_Toc209812199)

[Supplementary Tables 11](#_Toc209812200)

[Supplementary References 13](#_Toc209812201)

# Supplementary Text 1:

The Nile River Basin, spanning 11 countries and covering an area of approximately 3.2 million square kilometers, exhibits remarkable geographical and climatic diversity, resulting in highly variable hydrological conditions across its sub-basins. The basin's three primary tributaries and sub-basins (Fig. 1a) are the White Nile (WNS), Blue Nile (BNS), and Atbara (AS). The White Nile sub-basin originates from Lake Victoria, located in the equatorial region of East Africa, where annual rainfall exceeds 1600 mm (Fig. 1a and Supplementary Fig. 1a). This sub-basin includes several lakes, such as Kyoga and Albert, before the river flows through the vast Sudd wetlands in South Sudan, where approximately 50% of the water is lost to evaporation^1,2^. The White Nile subbasin lies in equatorial regions where temperatures remain above 30°C and receive bimodal rainfall patterns that provide year-round flow to the main Nile (Supplementary Fig. 1b).

The Blue Nile sub-basin originates from Lake Tana in the Ethiopian Highlands and contributes around 60% of the Nile’s total flow^2^. The Blue Nile has a short, intense rainy season, driven by the migration of the Intertropical Convergence Zone (ITCZ)^2^. The average annual rainfall ranges between 800 and 1,600 mm (Supplementary Fig. 1a), primarily occurring between June and September. The steep topography and concentrated rainfall result in rapid runoff and high seasonal discharge, leading to significant interannual variability in flow. The highlands experience cooler temperatures, with annual averages dropping below 15°C (Supplementary Fig. 1b). The Atbara sub-basin, the northernmost tributary of the Nile, channels runoff from the Ethiopian Highlands, much like the Blue Nile. The sub-basin experiences highly seasonal flows during the rainy season in August and September, but it remains dry during the rest of the year, where rainfall can be as low as 50 mm annually^2^ (Supplementary Fig. 1a).

Long-term hydrological records indicate that rainfall and river flow exhibit significant variability on annual to decadal scales, underscoring the importance of understanding the unique contributions of each tributary to the Nile’s overall water balance. Analysis of the stream flow data from the Mogren (WNS), Khartoum (BNS), and Kilo3 (AS) gauge stations for the period 1900 to 1999 revealed that the average contribution of the BNS, WNS, and AS to the downstream countries is 60.3%, 25.5%, and 14.2%, respectively^3^.

# Supplementary Text 2:

Statistical metrics for evaluating the performance of the SWAT+ model (Supplementary Table 1) indicate that Nash-Sutcliffe Efficiency (NSE) ranged from 0.80 to 0.87 for the calibration period and from 0.77 to 0.89 in the validation period, exceeding the >0.65 threshold for the “very good” performance designation^4,5^. The Percent Bias (PBIAS) remained between 8.01% and 14.12% for calibration and 7.46% and 9.71% for validation, within the “very good” to “good” range^6^. Kling-Gupta Efficiency (KGE) values spanned 0.71–0.87 in calibration and 0.70–0.90 in validation, indicating good to very good agreement^7^, confirming the model’s ability to capture spatial and temporal variability with minimal systematic bias across the key gauging stations.

# Supplementary Text 3:

Precipitation extremes were assessed using the annual maximum series (AMS) derived from CHIRPS (1984–2016) and bias-corrected GCM projections under SSP2-4.5 and SSP5-8.5 for 2025–2100. The AMS was computed as the maximum 30-day rolling precipitation total for each year, expressed in mm/30 days, to extract monthly-scale extremes and ensure consistency between climatic drivers (precipitation) and hydrologic responses (streamflow). The Log Pearson Type III Distribution (LP3), identified as the best fit for the AMS data, was applied to estimate precipitation intensities at return periods of 5–200 years. Uncertainty was quantified using the 5th and 95th percentiles of 1000 bootstrap resamples from the projected AMS of the three representative models under each scenario.

Supplementary Figure 4 presents the return period plots for precipitation extremes, comparing the historical baseline (1984–2016) with projections under SSP2-4.5 and SSP5-8.5 (2025–2100). The historical 5-, 10-, 25-, 50-, 100-, and 200-year return period precipitation extremes were estimated at 160, 169, 179, 186, 193, and 200 mm/30 days, respectively. Under SSP2-4.5 (50th percentile), these values are projected to increase to 209, 232, 260, 280, 298, and 316 mm/30 days, corresponding to rises of 31%, 38%, 45%, 50%, 54%, and 58%. Similarly, under SSP5-8.5 (50th percentile), the extremes increase further to 222, 256, 302, 338, 376, and 416 mm/30 days, reflecting growth of 39%, 52%, 68%, 81%, 95%, and 108% relative to the baseline.

These results highlight systematic increases in precipitation extremes across all return periods, with stronger amplification under the high-emission scenario (SSP5-8.5). Such consistent increases provide essential context for the amplified streamflow extremes reported in the main text.

**Supplementary Text 4:**

To evaluate the best-fitting probability distribution for extreme value analysis, six distributions were tested: Generalized Extreme Value (GEV), Generalized Pareto (GPA), Gumbel (GUM), 3-parameter Lognormal (LN3), log-Pearson Type III (LP3), and Pearson Type III (PE3). These distributions were applied to nine Annual Maximum Series (AMS) datasets for both streamflow and precipitation. The nine datasets were derived from three sources. The first was a historical dataset representing the 20th century. The second group included four datasets for the 21st century under the SSP2-4.5 scenario. These were based on projections from three climate models—ACCESS-ESM1-5 (low), MPI-ESM1-2-HR (medium), and CNRM-ESM2-1 (high)—along with an additional dataset created by randomly sampling from the three model outputs. Similarly, the third group consisted of four datasets for the SSP5-8.5 scenario in the 21st century, using ACCESS-ESM1-5 (low), GISS-E2-1-G (medium), and CNRM-ESM2-1 (high), plus one dataset from random sampling of these three. All distributions were fit to each of the nine datasets using the method of L-moments, a robust technique that reduces biases in skewed datasets^8^. The criteria to identify the best distribution was based on the three metrics: the ratio of L-moment parameters 3 (kurtosis) to 4 (skewness)^9^ and Anderson-Darling test results (test statistic and p-value). For each of the nine datasets, we ranked the six distributions from the best to the worst, from 1 to 6, and then added the nine scores together as the final score for each distribution based on one of three metrics. All the scores for six distributions are listed in Supplementary Table 3 (streamflow) and 4 (precipitation). The top three distributions (LP3, PE3, and GEV) have similar probability predictions (Supplementary Figure 6 (streamflow) and 7 (precipitation)). The LP3 had the best-fit score and was selected for streamflow and precipitation prediction at the return periods ranging from 5 to 200 years.

# Supplementary Figures

*a,* CHRIPS data shows the spatial distribution of average annual precipitation across the Nile Basin, ranging from 0.18 mm/year in hyper-arid regions to 2,780 mm/year in high-rainfall areas. *b,* CHRITS data shows the spatial distribution of average annual temperature, which varies between 11.5°C in the Ethiopian Highlands and 33.2°C in the arid northern regions.

Supplementary Fig. 1: Annual average Precipitation and Temperature map of Nile Basin


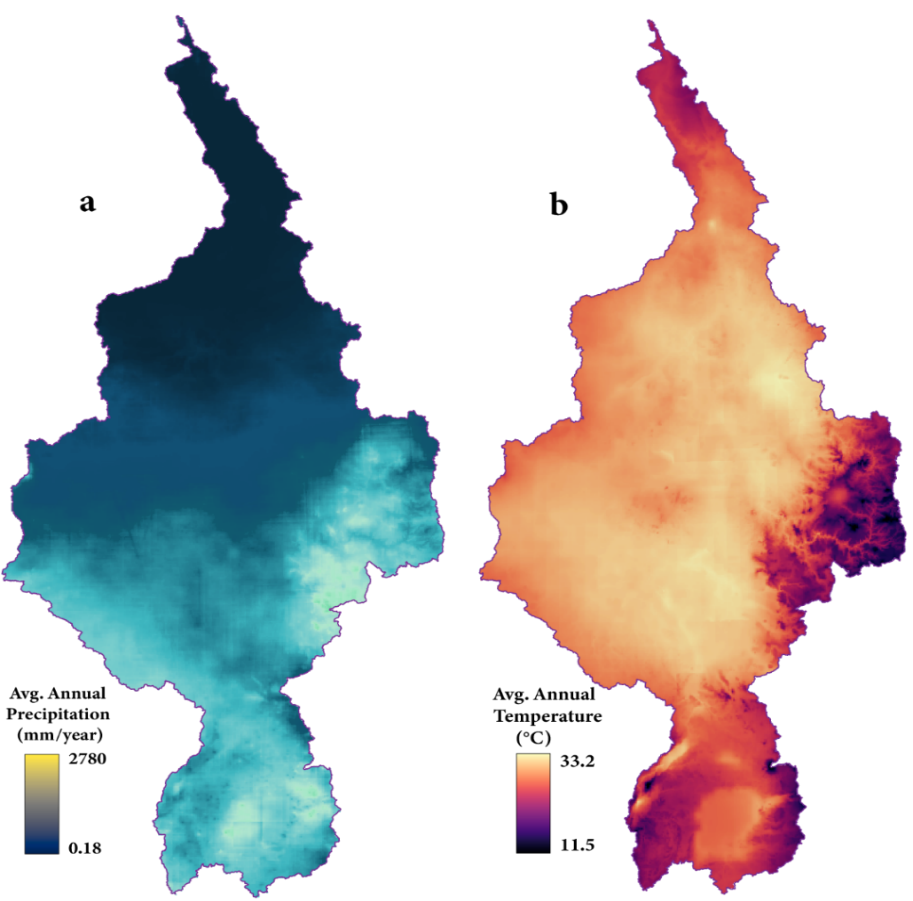


Supplementary Fig. 2: CDFs for observed, uncorrected and corrected model Precipitation data

The figure compares the upper-tail behavior of observed, uncorrected, and bias-corrected ACCESS-CM2 precipitation data under two different scenarios by displaying CDFs computed for the 90^th^ and 99^th^ percentile thresholds. This dual‐threshold analysis highlights how bias correction improves the model’s ability to capture both moderately and highly extreme precipitation events.


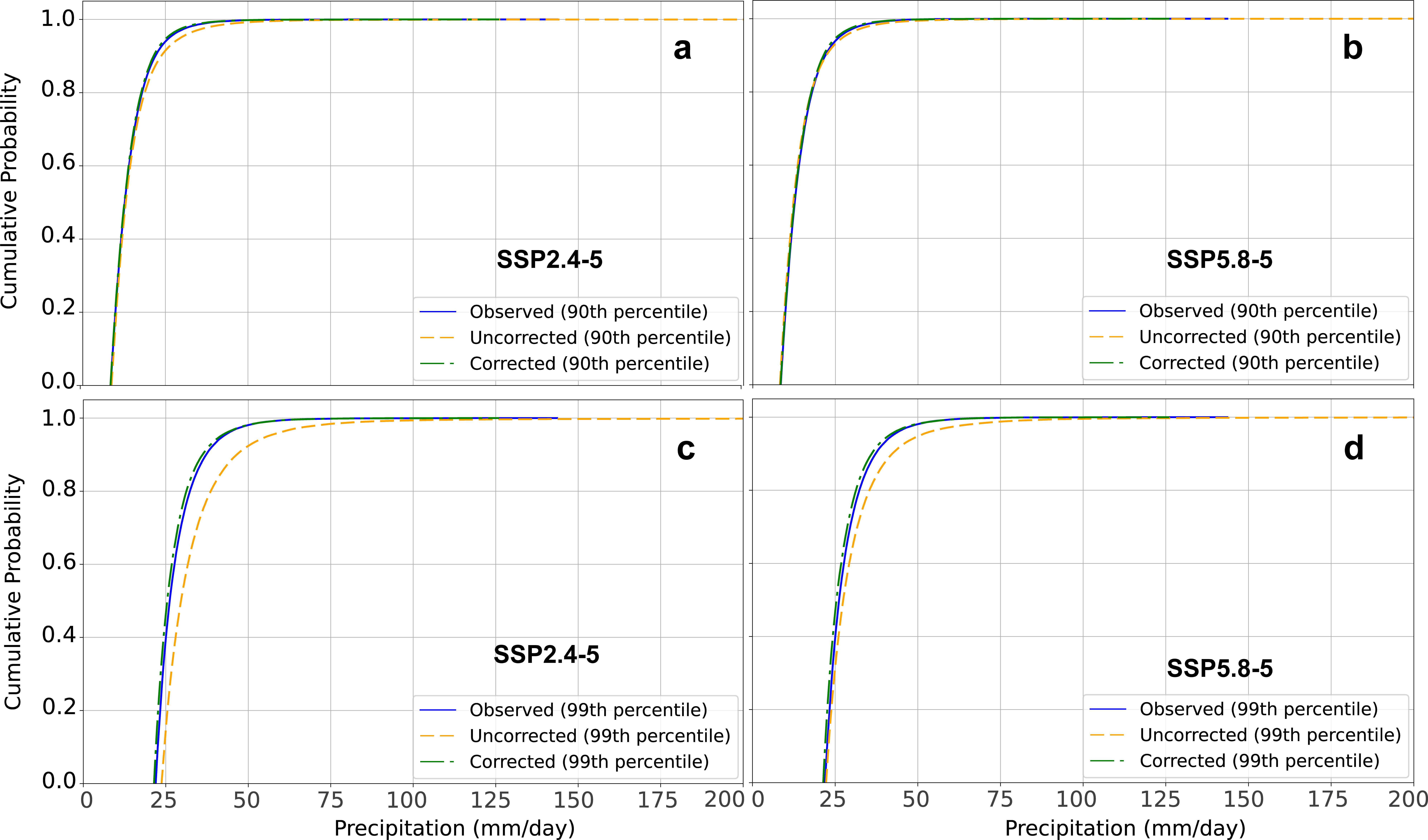


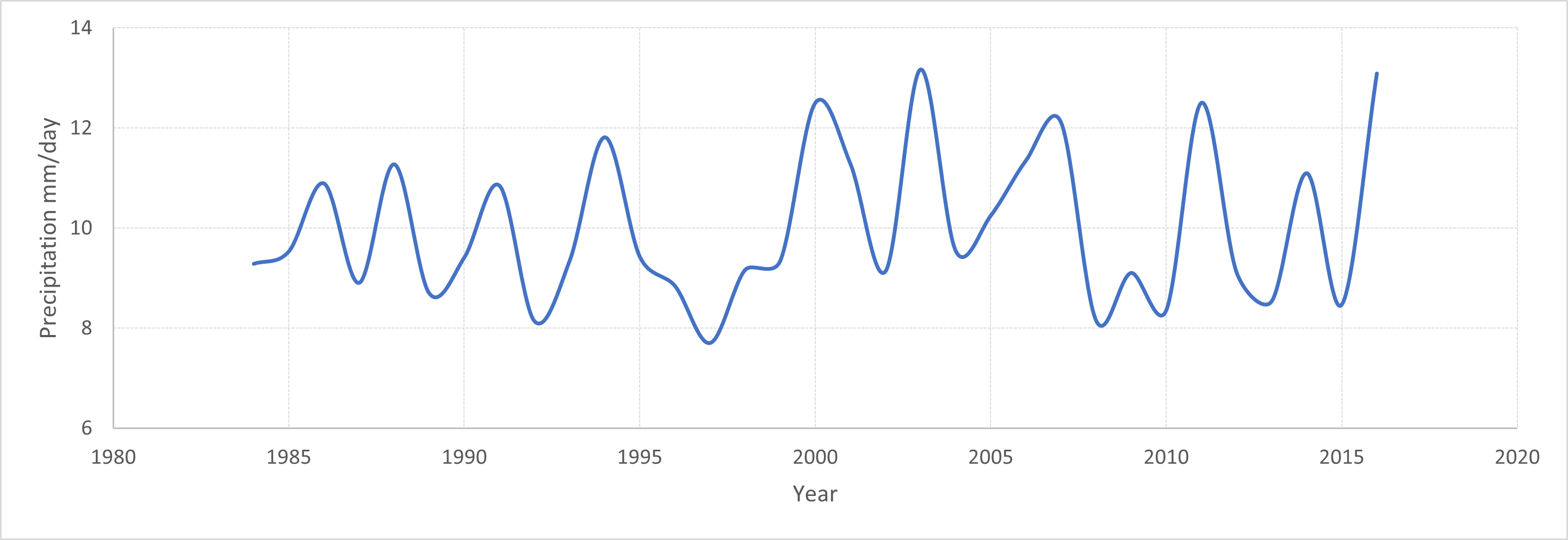


Supplementary Fig. 3: Observed Annual Maximum Precipitation (1984–2016)

Annual maximum daily precipitation (AMS) over the Nile Basin derived from CHIRPS observations during 1984–2016

Supplementary Fig. 4: Analysis for extreme precipitation at 30-day duration under SSP2-4.5 and SSP5-8.5 for the entire basin area contributing to the streamflow at the Dongola Station

Plots of precipitation extremes comparing precipitation intensity from the 20th century observations with projections for the 21st century under a, SSP2-4.5 and b, SSP5-8.5. The predicted extremes include precipitation intensity at return periods of 5, 10, 25, 50, 100, and 200 years, with the uncertainty represented by the 5th and 95th percentiles.


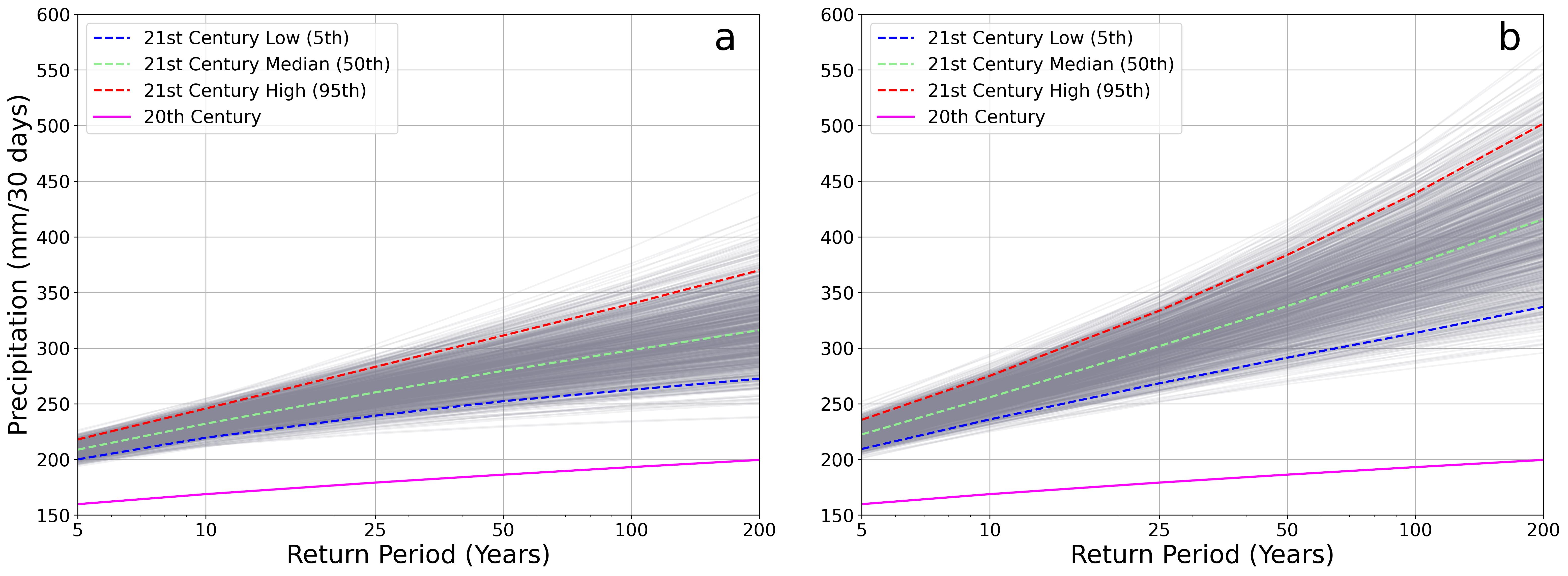


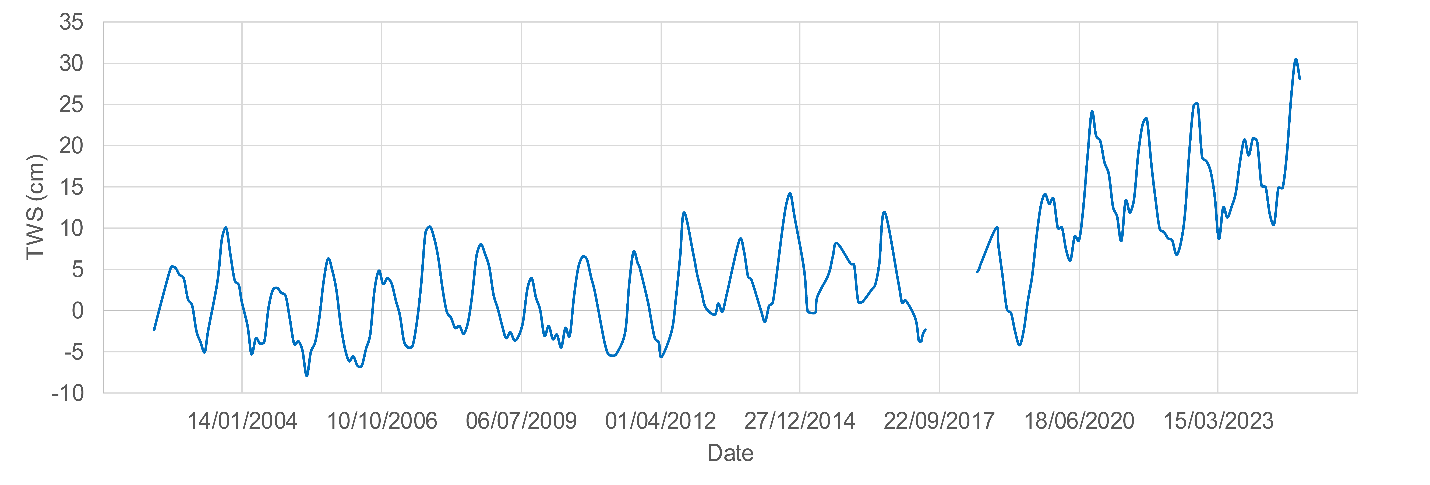


Supplementary Fig. 5: GRACE_TWS_ time series over the Nile basin.

A slight increase of 0.24 cm/year in the total water storage trend over the Nile Basin between 2002 and 2017 followed by larger increase of 2.4 cm/year between 2018 and 2024). The total water storage (TWS) was calculated using GRACE/GRACE-FO RL06.3 CSR Mascon data, by averaging monthly anomalies over the Nile basin^3,10,11^.

Supplementary Fig. 6: Predicted streamflow extremes at various return periods using six statistical distributions based on historical data (1984-2016)


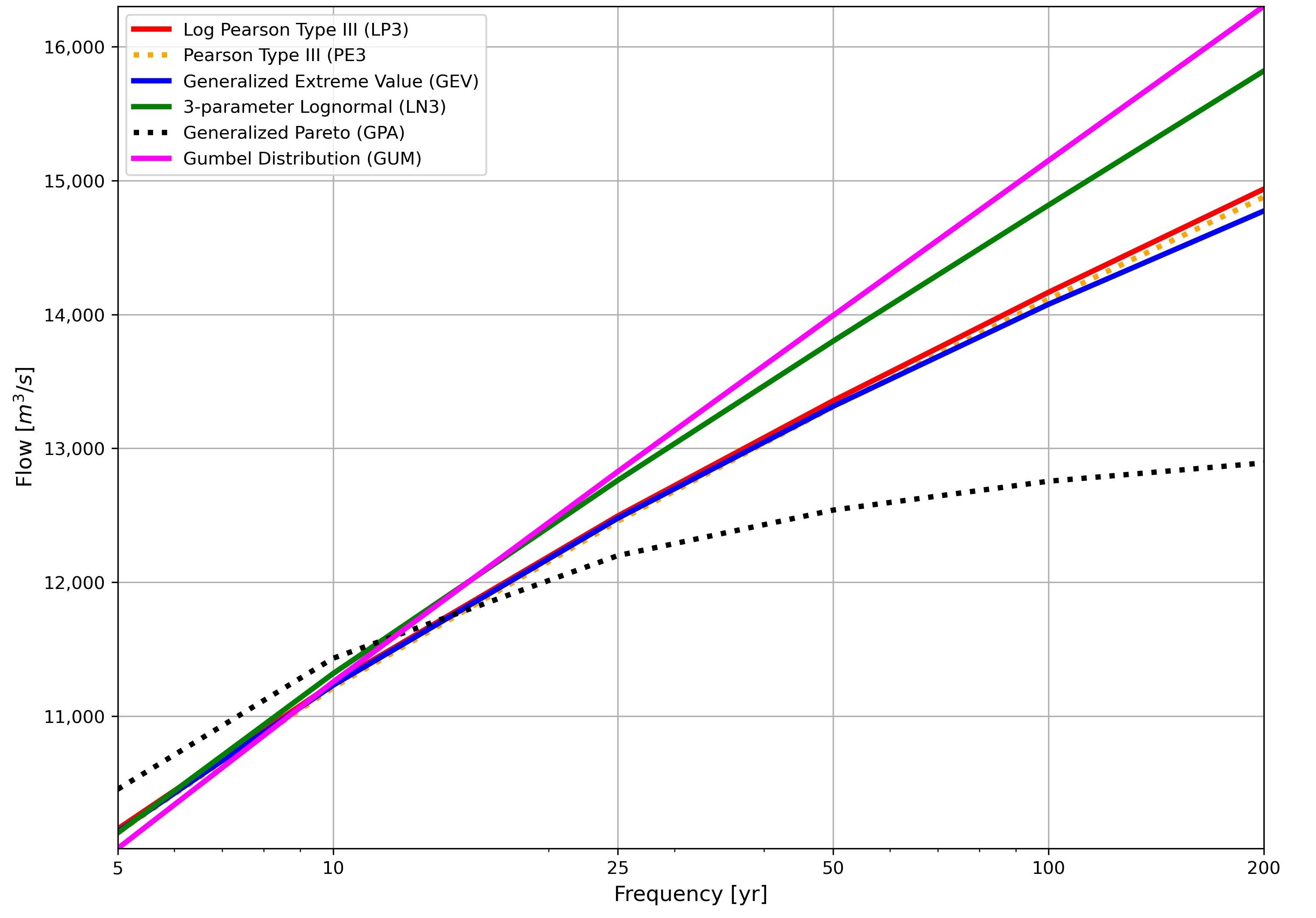


The figure compares predicted streamflows at various return periods (or frequencies) ranging from 5 to 200 years, using six different distributions based on the observation dataset. The GPA shows a low prediction for streamflow at high return periods and a high prediction at low return periods. LP3, PE3, and GEV have similar probability predictions; LP3 had the best-fit score and was selected for streamflow prediction at the return periods of 5 to 200 years.

Supplementary Fig. 7: Predicted precipitation extremes at various return periods using six statistical distributions based on historical data (1984-2016)


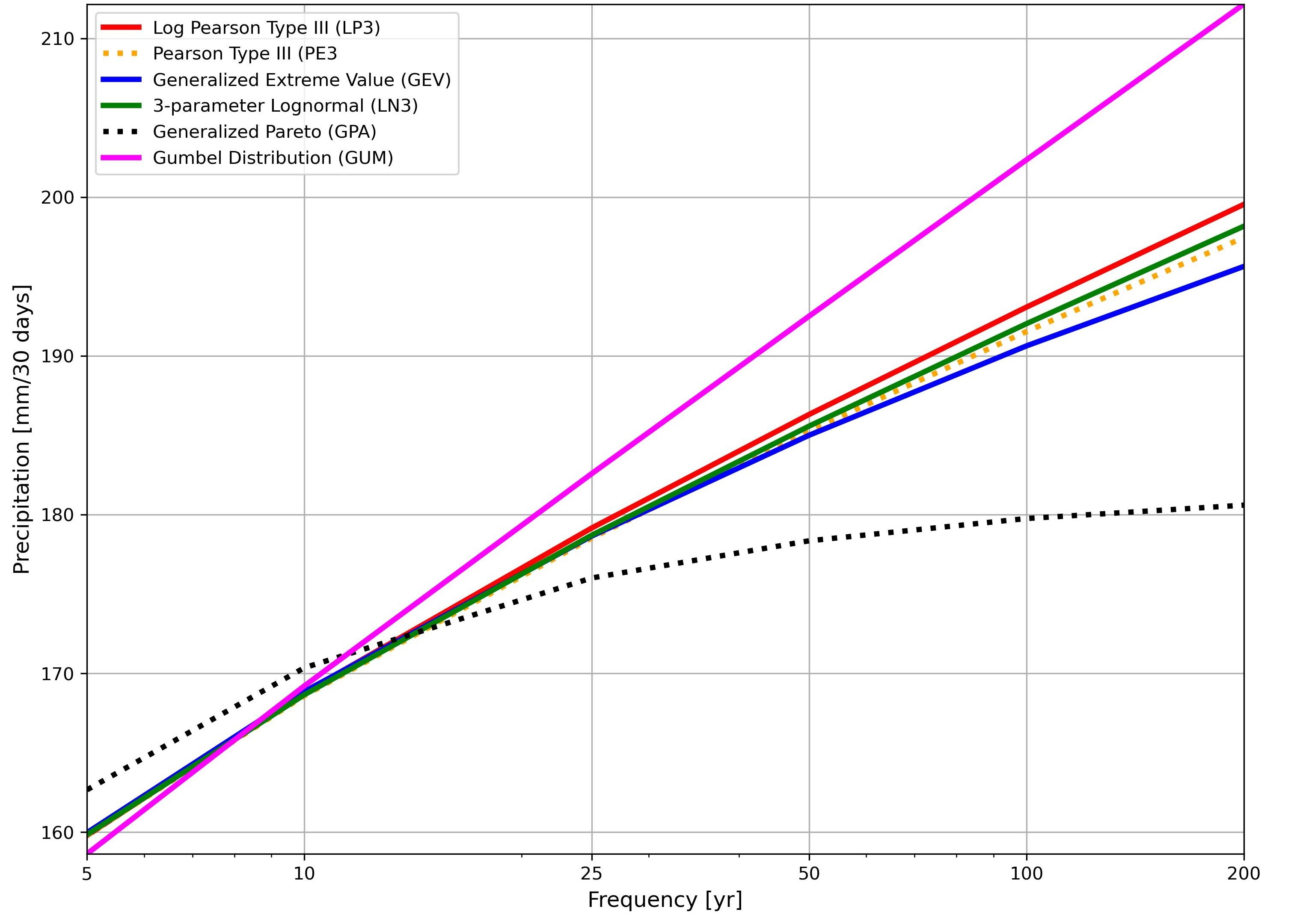


The figure compares predicted precipitation at various return periods (or frequencies) from 5 to 200 years with six different distributions based on the CHIRPS historical dataset (1984–2016). The GPA shows a low prediction of precipitation at high return periods and a high prediction at low return periods. GUM has a high prediction at high return periods and a low prediction at low return periods. GEV, LN3, PE3, and LP3 have similar probability predictions. LP3 had the best-fit score and was selected for precipitation prediction at return periods of 5 to 200 years.

# Supplementary Tables

| Supplementary Table 1. Performance metrics for the four stations during the calibration and validation periods. | | | | | | | | |
| --- | --- | --- | --- | --- | --- | --- | --- | --- |
| Metrics | Calibration | | | | Validation | | | |
|  | Dongola | Tamaniat | Khartoum | Sennar | Dongola | Tamaniat | Khartoum | Sennar |
| NSE | 0.84 | 0.8 | 0.87 | 0.86 | 0.89 | 0.77 | 0.86 | 0.88 |
| KGE | 0.75 | 0.71 | 0.87 | 0.83 | 0.90 | 0.74 | 0.70 | 0.73 |
| PBIAS | 14.12 | 8.01 | -6.20 | 1.2 | 7.46 | 9.71 | -25.6 | -24.83 |

| Supplementary Table 2. Source of the spatial and meteorological data for SWAT+ model. | | | | | |
| --- | --- | --- | --- | --- | --- |
| Data | Source | | Description | Year | Website |
| DEM | SRTM | Shuttle Radar Topography Mission at 90m resolution | | 2000 | <https://opentopography.org/> |
| GLCC | USGS | Global Land Cover Characterization (GLCC) at 1km resolution | | 1993 | <https://www.usgs.gov/media/images/global-land-cover-characteristics-data-base-version-20> |
| Soil | FAO-UNESCO | Global Soil Map at 1km resolution | | 2003 | <https://data.apps.fao.org/map/> |
| Projected Climate | CMIP6 | \| Downscaled CMIP6 climate projections at 0.25º resolution \| \| --- \| | | 2015–2100 | <https://www.nccs.nasa.gov/services/data-collections/land-based-products/nex-gddp-cmip6> |
| Historical Precipitation & Temperature | CHRIPS & CHRITS | Precipitation and Temperature data at 0.25º resolution | | 1983–2016 | <https://data.chc.ucsb.edu/products/CHIRPS-2.0/>  <http://data.chc.ucsb.edu/products/CHIRTSdaily/> |
| Historical Solar radiation, Wind speed, Relative humidity | CFSR | Global reanalysis dataset for meteorological parameters at 0.5º resolution | | 1983–2016 | <https://climatedataguide.ucar.edu/climate-data/climate-forecast-system-reanalysis-cfsr> |
| Terrestrial Water Storage (TWS) | CSR | GRACE & GRACE-FO | | 2002-2024 | https://www2.csr.utexas.edu/grace/ |

Supplementary Table 3. Goodness-of-fit ranking for probability distributions tested for streamflow.

| Distribution | Anderson–Darling Test | | L-Moment Parameter Ratio Score | Cumulative Score |
| --- | --- | --- | --- | --- |
|  | P-Value Score | Test Statistic Score |  |  |
| log Pearson Type III (LP3) | 21 | 24 | 12 | 57* |
| Pearson Type III (PE3) | 17 | 22 | 27 | 66 |
| Generalized Extreme Value (GEV) | 20 | 16 | 31 | 67 |
| Gumbel Distribution (GUM) | 32 | 29 | 52 | 113 |
| Generalized Pareto (GPA) | 48 | 45 | 32 | 125 |
| 3-parameter Lognormal (LN3) | 51 | 54 | 35 | 140 |

* The lowest cumulative score represents the best-fit score.

Supplementary Table 4. Goodness-of-fit ranking for probability distributions tested for precipitation.

| Distribution | Anderson–Darling Test | | L-Moment Parameter Ratio Score | Cumulative Score |
| --- | --- | --- | --- | --- |
|  | P-Value Score | Test Statistic Score |  |  |
| log Pearson Type III (LP3) | 17 | 23 | 19 | 59* |
| Pearson Type III (PE3) | 14 | 28 | 27 | 69 |
| Generalized Extreme Value (GEV) | 25 | 20 | 26 | 71 |
| Gumbel Distribution (GUM) | 34 | 19 | 54 | 107 |
| Generalized Pareto (GPA) | 45 | 45 | 36 | 126 |
| 3-parameter Lognormal (LN3) | 54 | 54 | 35 | 143 |

* The lowest cumulative score represents the best-fit score.

Supplementary Table 5. List of the 30 GCMs from NEX-GDDP used in this study.

| # | GCM | # | GCM |
| --- | --- | --- | --- |
| 1 | ACCESS-CM2 | 16 | GISS-E2-1-G |
| 2 | ACCESS-ESM1-5 | 17 | IITM-ESM |
| 3 | BCC-CSM2-MR | 18 | INM-CM4-8 |
| 4 | CESM2 | 19 | INM-CM5-0 |
| 5 | CESM2-WACCM | 20 | IPSL-CM6A-LR |
| 6 | CMCC-CM2-SR5 | 21 | KIOST-ESM |
| 7 | CMCC-ESM2 | 22 | MIROC-ES2L |
| 8 | CNRM-CM6-1 | 23 | MPI-ESM1-2-HR |
| 9 | CNRM-ESM2-1 | 24 | MPI-ESM1-2-LR |
| 10 | CanESM5 | 25 | MRI-ESM2-0 |
| 11 | EC-Earth3 | 26 | NESM3 |
| 12 | EC-Earth3-Veg-LR | 27 | NorESM2-LM |
| 13 | FGOALS-g3 | 28 | NorESM2-MM |
| 14 | GFDL-CM4 | 29 | TaiESM1 |
| 15 | GFDL-ESM4 | 30 | UKESM1-0-LL |

# Supplementary References

1. Conway, D. & Hulme, M. Recent fluctuations in precipitation and runoff over the Nile sub-basins and their impact on main Nile discharge. Clim. Change 25, 127–151 (1993).
2. Sutcliffe, J. V. & Parks, Y. P. The Hydrology of the Nile (International Association of Hydrological Sciences, 1999).
3. Badawy, A., Sultan, M., Abdelmohsen, K., Yan, E., Elhaddad, H., Milewski, A. & Torres-Uribe, H. E. Floods of Egypt’s Nile in the 21st century. Sci. Rep. 14, 27031 (2024).
4. Moriasi, D. N., Arnold, J. G., Van Liew, M. W., Bingner, R. L., Harmel, R. D. & Veith, T. L. Model evaluation guidelines for systematic quantification of accuracy in watershed simulations. *Trans. ASABE* **50**, 885–900 (2007).
5. Saleh, A., Arnold, J. G., Gassman, P. W., Hauk, L. M., Rosenthal, W. D., Williams, J. R. & MacFarland, A. M. S. Application of SWAT for the Upper North Bosque River watershed. Trans. ASAE 43, 1077–1087 (2000).
6. Van Liew, M. W., Veith, T. L., Bosch, D. D. & Arnold, J. G. Suitability of SWAT for the Conservation Effects Assessment Project: Comparison on USDA Agricultural Research Service watersheds. J. Hydrol. Eng. 12, 173–189 (2007).
7. Gupta, H. V., Kling, H., Yilmaz, K. K. & Martinez, G. F. Decomposition of the mean squared error and NSE performance criteria: Implications for improving hydrological modelling. J. Hydrol. 377, 80–91 (2009).
8. Hosking, J. R. M. L-moments: Analysis and estimation of distributions using linear combinations of order statistics. J. R. Stat. Soc. B 52, 105–124 (1990).
9. Hosking, J. R. M. & Wallis, J. R. Some statistics useful in regional frequency analysis. *Water Resour. Res.* 29, 271–281 (1993).
10. Abdelmohsen, K., Sultan, M., Save, H., Abotalib, A. Z. & Yan, E. What can the GRACE seasonal cycle tell us about lake–aquifer interactions? Earth-Sci. Rev. 211, 103392 (2020).
11. Elhaddad, H., Sultan, M., Yan, E., Abdelmohsen, K., Mohammad, A. T., Badawy, A., Karimi, H., Saleh, H. & Emil, M. K. Optimization of floodwater redistribution from Lake Nasser could recharge Egypt’s aquifers and mitigate its excessive floods. Commun. Earth Environ. 5, 385 (2024).
